# Possibilities for Efficient Furniture Construction Made of Thin and Ultra-Thin Materials by Using Mitre Joints

**DOI:** 10.3390/ma16216855

**Published:** 2023-10-25

**Authors:** Boryana Petrova, Vassil Jivkov, Nikolay Yavorov

**Affiliations:** 1 Department of Interior and Furniture Design, Faculty of Forest Industry, University of Forestry, 1797 Sofia, Bulgaria; b.petrova@ltu.bg; 2 Department of Interior and Architectural Design, Faculty of Architecture, University of Architecture, Civil Engineering and Geodesy, 1046 Sofia, Bulgaria; 3 Department of Pulp, Paper and Printing Arts, University of Chemical Technology and Metallurgy, 1756 Sofia, Bulgaria; nyavorof@uctm.edu

**Keywords:** mitre joints, thin materials, ultra-thin materials, bending moment, stiffness, wood-based materials, non-wood materials, plywood, MDF, compact HPL

## Abstract

One of the biggest challenges for designers and manufacturers of furniture is to reduce the thickness of conventional furniture materials such as particleboard (PB), medium-density fibreboard (MDF) and plywood. Designing furniture based on thin (less than 16 mm) and ultra-thin materials (less than 10 mm) is desirable for aesthetic reasons and because of the substantial material savings. However, the use of thin and ultra-thin materials reduces the strength of the furniture, especially the strength and deformation resistance of the joints. This study aimed to establish the possibilities for efficient furniture construction made of thin and ultra-thin materials using mitre joints. For this purpose, 14 types of L-type joints were tested: 12 glued and 2 detachable. The joints were made of eight wood-based panels and one non-wood panel. The bending moments and the stiffness coefficient under compression were determined. The obtained results show that the mitre joints made of laminated material with high-pressure laminate (HPL), 8 mm thick, MDF achieved the highest bending moment, and the highest stiffness coefficient was achieved by joints made of 10 mm thick compact HPL. Compact HPL joints were significantly affected by the type of adhesive used. Detachable joints had a relatively high bending strength but very low stiffness.

## 1. Introduction

Wood and wood-based materials are the main materials used in furniture construction. They are preferable due to their many advantages, such as a natural and unique wood texture and a good strength–weight ratio. Moreover, they are produced from renewable raw materials suitable for recycling, thus making them particularly suitable as an object of a circular economy. On the other hand, the increased demand for wood and wood products makes this raw material valuable and desirable. This requires their optimal use.

The main wood-based materials used in furniture construction are particleboard (PB), medium-density fibreboard (MDF), plywood, a combination of all three and solid wood. Most often, panels with 16, 18 or 19 mm thicknesses are used for case furniture. For example, in eastern European countries, a thickness of 18 mm is mainly used. In central and western European countries, 16 mm is used for kitchens and 19 mm for wardrobes and other cupboard furniture. Reducing the thickness of the panels used in furniture construction from 16 mm to 12 mm leads to a savings of 25%, from 18 to 12 mm provides 33% and from 19 mm to 9 mm provides almost 47%.

On the other hand, design trends have led to a reduction in the thickness of structural elements. In the search for elegant and clean lines, designers are increasingly reducing the thickness of structural elements [1]. Thinner materials also reduce transport and storage costs, and furniture made from thinner materials is lighter and provides performance advantages for the end user [2].

One issue in the design of furniture made of thin materials is the adequate choice of suitable material. This is especially valid for wood-based materials. According to Jivkov and Elenska-Valchanova [3], structural wood-based furniture materials are categorised into five groups according to their structural–technological properties: ultra-thin—less than 10 mm; thin—10–15 mm; standard—16–19 mm; thick—20–40 mm; ultra-thick—more than 40 mm. Thin and ultra-thin structural elements of PB, plywood or MDF are often used for drawer construction elements such as drawer bottoms or furniture backs. Contemporary furniture design usually requires more novel and unconventional design solutions, such as whole furniture made of thin and ultra-thin materials. In these cases, data on their strength characteristics are essential. Studies on the properties of thin and ultra-thin furniture panels can be found in the specialised literature. Mainly, these are studies of plywood [4,5,6,7,8], PB [5,9], MDF [3,5,8,9,10,11,12,13], oriented strand board (OSB) [9] or a combination of some of them [3]. Some authors are looking at possibly reinforcing wood-based panels to increase strength and deformation properties, such as with thin aluminium sheets [14].

A significant problem in using thin, especially ultra-thin, panel materials is providing reliable joints with the required strength and deformation characteristics. There are relatively few data in the literature on the strength characteristics of joints of thin structural elements. Extensive research on the strength and deformation characteristics of end-corner joints made of thin wood-based panel structural elements has been conducted by Langova and Joscak [15,16]. They investigated the strength and stiffness of corner joints using Confirmat with the dimensions ø5 × 40, ø5 × 50, ø7 × 50 and ø7 × 70 mm in MDF and PB structural elements with thicknesses of 12 and 18 mm. The lowest bending moment at 12 mm thickness is achieved by the ø5 × 40 mm Confirmat joints made of PB structural elements (3.63 N·m), and the highest is achieved with ø7 × 70 mm Confirmat in MDF (11.31 N·m). The highest stiffness coefficient was found for MDF joints with ø7 × 70 mm Confirmat (184.92 N·m/rad) and the lowest for PB joints with ø5 × 50 mm Confirmat (73.31 N·m/rad). Based on the results, it is concluded that the parameters, especially the screw body diameter, thickness and density of the material, significantly affect the stiffness and load-carrying capacity of the corner joints.

In 2019, Machova et al. [17] investigated the effect of atmospheric humidity on the strength and deformation characteristics of end-corner joints made of 12 mm thick PB. Glued joints by dowels (ø6 mm) and detachable joints by eccentric connectors and non-glued dowels (ø6 mm) were investigated. For the glued joints by dowels at room temperature and an atmospheric humidity of 90%, they found that the maximum bending moment is 3.88 N·m and the stiffness coefficient is 231.89 N·m/rad. For the detachable joints, the corresponding values are 2.26 N·m and 134.99 N·m/rad. The authors found that as the atmospheric humidity decreased from 90% to 45% at a temperature of 23 °C, the strength and deformation characteristics of the joints were also reduced. The strength of the glued joints by dowels decreased by 38%, at an atmospheric humidity of 45% and a temperature of 23 °C, reaching relatively low values of 2.40 N·m. It should be noted that four 6 mm diameter dowels are used for these joints. The difference at different humidity is only 6% for the detachable joints.

A team of researchers investigated the influence of the type of furniture panel and its thickness on the strength and stiffness of screw joints in cabinet furniture construction [18]. The materials studied were PB with thicknesses of 16 and 18 mm, MDF with thicknesses of 16 and 18 mm, and plywood from Okoume veneer (Aucoumea klaineana) with a thickness of 15 mm. They concluded that the strength of the joint made of plywood is higher than that of the 18 mm thick PB and MDF. They drew the same conclusion about the stiffness of the joints. The joints made of 15 mm thick plywood structural elements showed bending moments of 79.61 N·m when loaded in the arm compression test and 99.00 N·m when in the arm opening test. 

Yuksel et al. [19] performed a similar study as above but included the Confirmat and Minifix connectors in addition to screws. Again, the joints made of plywood structural elements had the highest strength in all three joining options and outperformed those with 18 mm thick PB and MDF. When the Minifix joints made of 15 mm thick structural elements were loaded in the arm compression test, the bending moment was 49.05 N·m with two connectors per joint. The bending moments for Confirmat and wood screws are 70.14 N·m for both types of connectors.

In another study of end-corner joints with a Minifix connector, it was found that reducing the thickness of particleboard from 16 to 12 mm resulted in a reduction in bending strength of 34% for the arm compression test and 48% for the arm opening test [20]. 

Lamello-type wooden biscuits are one of the popular options for achieving reliable and durable joints in furniture. In the scientific literature, there have been several publications that have focused on establishing the strength and deformation characteristics of Lamello joints mainly made of particleboard and MDF [21,22,23,24,25,26,27], but all of them are for panels with thicknesses of 16 mm or above.

Among the joints traditionally defined as highly resistant joints are mitre joints [28]. It makes them suitable for use in thin, especially ultra-thin, furniture structures. However, relatively few studies have been conducted to establish mitre joints’ strength and deformation characteristics [21,29,30], and we found no studies conducted with thin or ultra-thin wood-based materials. Norvydas et al. [30] concluded that adhesive bonding of the mitre joint appears to be the most convenient method for joining panels of wood and PB.

Some traditional non-wood materials for the furniture industry can also be used as an alternative to wood-based materials, such as compact HPL [31]. For compact HPL, no information was found on its joints’ strength or deformation characteristics. 

As a result of this review, it can be concluded that there is a lack of research on the strength and deformation characteristics of joints made of thin materials. Using an analysis of variance, this article revealed the possibilities of constructing furniture from thin or ultra-thin wood-based and non-wood materials using mitre joints. The aim of this study was to establish the possibilities for efficient furniture construction made of thin and ultra-thin materials using mitre joints. For this purpose, 14 types of joints were tested, of which 12 were glued and 2 were detachable. The joints are made of eight wood-based panels and one non-wood panel.

## 2. Materials and Methods

### 2.1. Materials

In this work, the three most popular wood-based materials for furniture were selected: plywood (9 and 12 mm), MDF (10 and 12 mm) and laminated PB (12 mm). Additionally, 9 mm thick plywood and 10 mm thick MDF were available with an HPL face layer. Of the non-wood materials, we selected 10 mm thick compact HPL. This decision was based on the fact that this material can be processed with the woodworking machines used in the furniture industry. This puts it technologically in the same group as wood-based materials. All materials are commercially produced. Detailed descriptions of the materials, their physical and mechanical characteristics and testing methods are given in a previous study by the authors [5].

**Figure 1:**
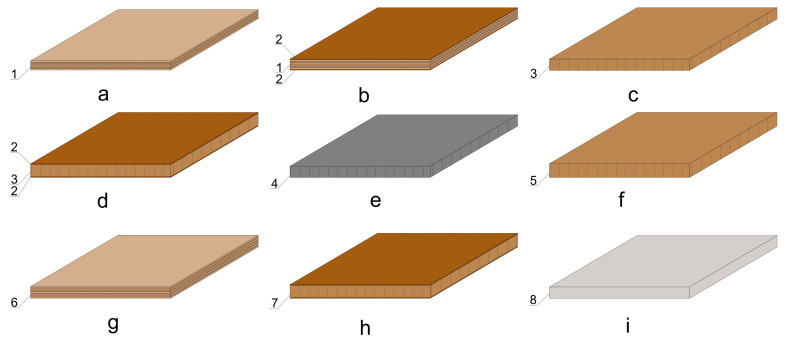
Types of thin and ultra-thin structural wood-based and non-wood materials: (**a**)—9 mm birch veneer plywood (1); (**b**)—9 mm birch veneer plywood (1) laminated with 0.8 mm thick high-pressure laminate (2) (HPL); (**c**)—10 mm medium-density fibreboard (3) (MDF); (**d**)—10 mm MDF (3) laminated with 0.8 mm HPL (2); (**e**) —10 mm black MDF (4); (**f**)—12 mm MDF (5); (**g**)—12 mm plywood with birch face veneer (6); (**h**)—12 mm laminated particleboard (7) (LPB); and (**i**)—10 mm compact HPL (8).

All materials included in this study are shown graphically in Figure 1. Data about the density (D), thickness (T), modulus of elasticity (MOE) and bending strength (BS) of thin and ultra-thin structural wood-based and non-wood materials are given in Table 1. For the plywood, the data are provided for the modulus of elasticity and bending strength in the transverse directions of the wood fibres of the face veneer.

### 2.2. Type of Joints

**Figure 2:**
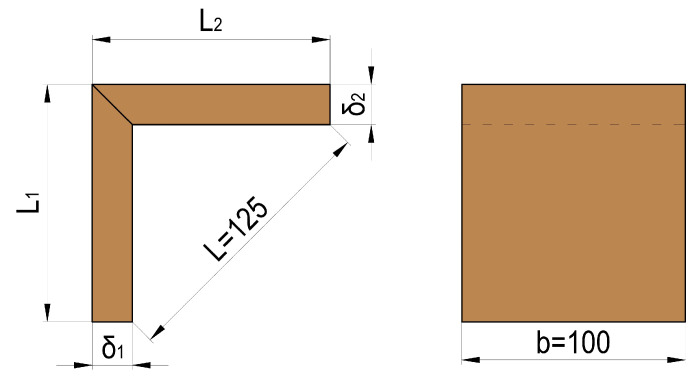
Type and dimensions of the tested samples.

An L-type joint was selected for the purpose of this study. The type of joints and dimensional parameters of the test samples, made in accordance with the testing method described by Kyuchukov and Jivkov [28], are shown in Figure 2. The dimensions δ_1_ and δ_2_ are equal and correspond to the thickness of the panels. The dimensions L_1_ and L_2_ are also equal and depend on the thickness of the panels. For each series of joints, 15 test samples were made. 

**Figure 3:**
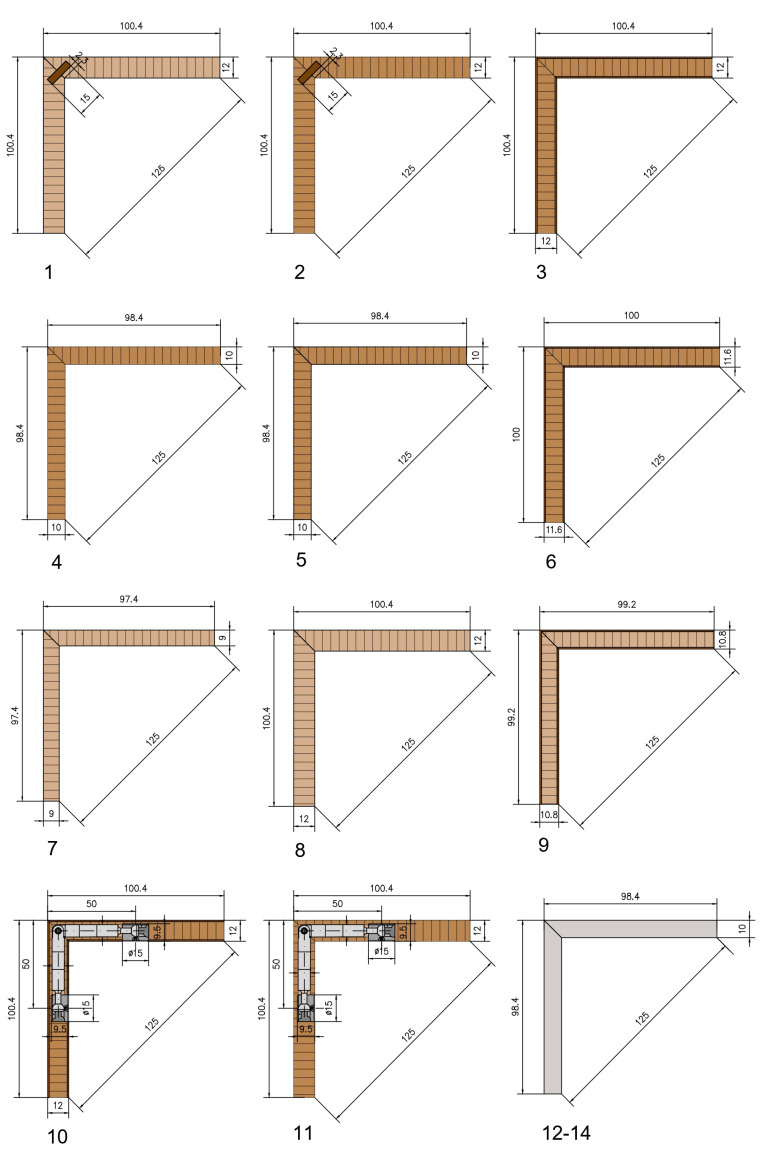
Mitred corner joints made of thin and ultra-thin panels: (**1**)—12 mm plywood joint by wooden biscuit (1_Ply_12 _L0); (**2**)—12 mm MDF joint by wooden biscuit (2_MDF_12 _L0); (**3**)—12 mm LPB with PVAc (3_LPB_12); (**4**)—10 mm MDF with PVAc (4_MDF_10); (**5**)—10 mm black MDF with PVAc (5_MDF_10_Black); (**6**)—10 mm laminated MDF with PVAc (6_MDF_10_HPL); (**7**)—9 mm plywood with PVAc (7_Ply_9); (**8**)—12 mm plywood with PVAc (8_Ply_12); (**9**)—9 mm laminated plywood with PVAc (9_Ply_9_HPL); (**10**)—12 mm LPB with Minifix (10_LPB_12_M_15); (**11**)—12 mm plywood with Minifix (11_MDF_12_M_15); (**12**)—10 mm compact HPL with Soudal SoudaBond Easy (12_HPL_10_Comp); (**13**)—10 mm compact HPL with Bison Grizzly Extreme (13_HPL_10_Comp); and (**14**)—10 mm compact HPL Bison Montage Kit (14_HPL_10_Comp).

Descriptions of L-type corner joints made of thin and ultra-thin structural elements, including type of material, thickness of the panels, face material where available, total thickness and connecting element or type of adhesive, are given in Table 2. The index, shown in the last column of Table 2, was used to interpret the results for a brief description of the type of joint. Figure 3 shows all the specific dimensions of the joints.

The adhesive used for the glued wooden joints is polyvinyl acetate (PVAc) adhesive of the German company Kleiberit (Weingarten, Germany)—PVAc Kleiberit 301.2, class D3. The tensile strength of PVAc adhesive, according to [32], is 4.71 N/mm^2^. The amount of applied adhesive is 180–220 g/m^2^. The joints’ structural elements were bonded at room temperature (20 ± 2 °C) and an atmospheric humidity of 50 ± 5%. 

Two of the joints (1_Ply_12 _L0 and 2_MDF_12 _L0) were made with wooden biscuits (Figure 3(1,2)). They were made with Lamello 0, with dimensions 47 mm × 15 mm × 4 mm, from beech wood, produced by the Swiss company Lamello AG (Bubendorf, Switzerland). All joints were glued only in the groove area of connecting elements. Each joint had only one wooden biscuit as a connecting element, placed in the middle of the width of the test sample. Seven mitre joints made of thin and ultra-thin panels were only glued (Figure 3(3–9)). Two detachable joints (10_LPB_12_M_15 and 11_MDF_12_M_15) were made with a Minifix connector with a housing of ø15 mm and a drilling depth of 9.5 mm (Figure 3(10,11)). The bolt was double ended for double-sided installation, and mitre angles ranged from 90° to 180° with a length of 44 mm. Connectors were produced by the German company Häfele SE & Co. KG (Baden-Württemberg, Germany). Each joint had only one Minifix connector, placed in the middle of the width of the test sample. Three joints were made of 10 mm compact HPL (Figure 3(12–14)). Different types of adhesives were used to bond the compact HPL joints (Figure 3(12–14)). The first one (12_HPL_10_Comp) was Soudal SoudaBond Easy (chemical base: polyurethane foam), produced by Soudal Group, Turnhout, Belgium with a tensile strength (DIN EN 1607 [33]) of 0.19 N/mm^2^ and a shear strength (DIN EN 12090 [34]) of 0.142 N/mm^2^. The second one (13_HPL_10_Comp) was Bison Grizzly Extreme (chemical base: shape-memory polymer), produced by Bolton Adhesives, Rotterdam, The Netherlands, with a tensile strength (DIN EN 1607) of 1.8 N/mm^2^ and a shear strength (DIN EN 12090) of 2.5 N/mm^2^. The third one (14_HPL_10_Comp) was the Bison Montage Kit (chemical base: acrylic dispersion), produced by Bolton Adhesives, The Netherlands, with a shear strength (DIN EN 12090) of 6 N/mm^2^. 

### 2.3. Test Methods

**Figure 4:**
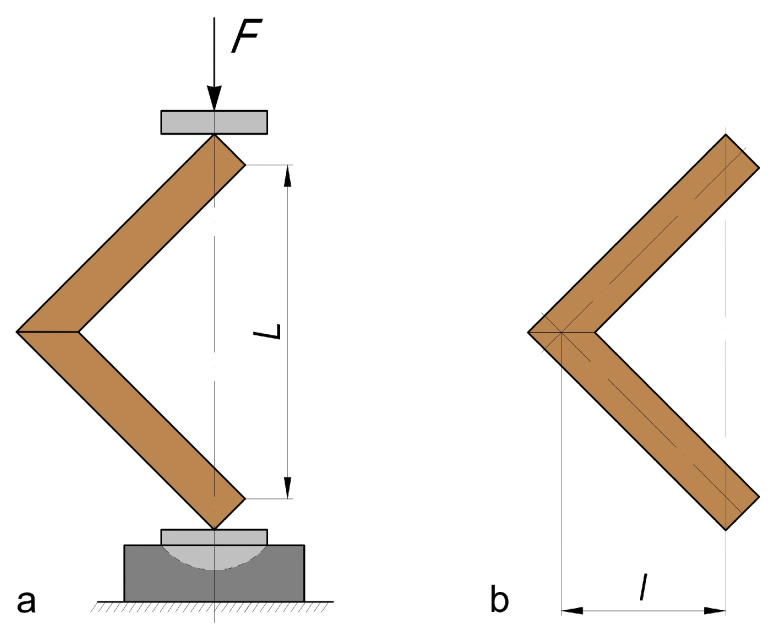
Test procedure [28]: (**a**) type of loading of the tested samples; (**b**) determination of the bending arm.

**Figure 5:**
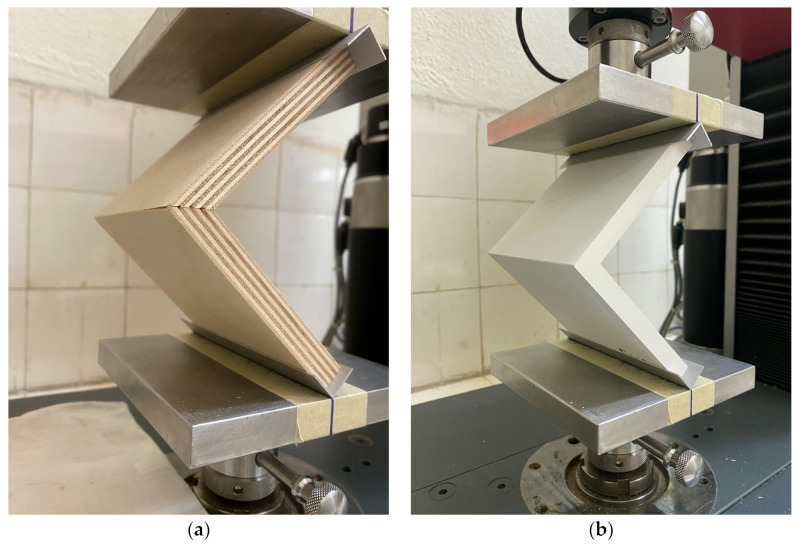
Test samples during testing: (**a**) test sample made of plywood; (**b**) test sample made of compact HPL.

The general test scheme and bending arm determination are presented in Figure 4. Figure 5 shows a test specimen during testing on a universal testing machine.

The criterion for determining the strength of the tested joints is the maximum bending moment, Mmax, calculated according to the following formula:

Mmax = F . l,(1)

where F is the maximum force under arm compression bending, N, and l is the arm, m.

The stiffness coefficient, c, is the criterion for determining the deformation characteristic of the corner joints [28,35].

**Figure 6:**
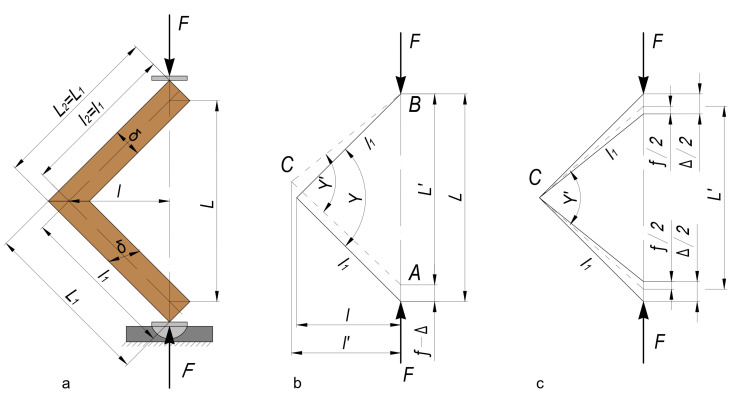
Test scheme and deformation under arm compression bending load of the test samples of end-corner joints made of lightweight panels [28]: (**a**) type of loading and dimensions of the tested samples; (**b**) scheme of loading; (**c**) determination of the deformation of the tested samples.

The deformation of the joints under the compression bending test causes changes in both the right angle between the joint arms and the bending arm, l, of the forces (Figure 6).

The linear displacement, *f_i_*, of the application points of the forces, *F_i_*, is recorded for each test sample at each loading level. It represents a sum of displacement resulting from turning the joint arms and additional displacement, Δ*_i_*, resulting from bending of the arms.

The displacement, Δ*_i_*, is calculated by the following formula:

(2) $$\Delta_{i}=\frac{F_{i}a^{3}}{3EI},$$

where *F_i_* is the magnitude of the load forces with arm compression, *N*; *a* is the axial length of the joint arms, *m*; *E* is the modulus of elasticity, N/mm^2^; and *I* is the axial moment of inertia of the cross-section of the joint arms, *m*^4^, which is calculated by the following formula:

(3) $$I=\frac{\delta b^{3}}{12},$$

where *b* is the width of the arms, *m*, and *δ* is the thickness of the arms, *m*.

The formula determines the distance between the force application points at each level of loading:

(4) $$L_{1}=L-f_{i}+\Delta_{i},$$

The angle $\gamma_{i}$ (rad) changed under loading between the joint arms is calculated by the following formula:

(5) $$\gamma_{i}=2\arcsin\frac{L_{i}}{2a}=2\arcsin\frac{L-f_{i}+\Delta_{i}}{2a},$$

The changed bending arm, *l_i,_* is determined by the following formula:

(6) $$l_{i}=a\cos\frac{\gamma_{i}}{2},$$

The result from the deformation under the compression bending test is the semi-rigid rotation of the joint arms in radians(rad):

(7) $$\alpha_{i}=\frac{\pi }{2}-\gamma_{i},$$

For 10 and 40% of the load force, *F_i_*, the bending moment in N·m is calculated according to the following formula:

(8) $$M_{i}=F_{i}l_{i},$$

The stiffness coefficient under the compression bending test, *c_i_* (N·m/rad), is calculated by the following formula:

(9) $$c_{i}=\frac{\Delta M_{i}}{\Delta \alpha_{i}},$$

In (8), the following designations are used:

$$\Delta M_{i}=M_{i}-M_{0}$$

$$\Delta \alpha_{i}=\alpha_{i}-\alpha_{0}$$

where *M_i_* and *α_i_* are determined according to (8) and (7), respectively, for the value of force, *F_i_*, equal to 40% of *F_max_*, and *M*_0_ and *α*_0_ are determined according to (8) and (7) or the value of force, *F*_0_, equal to 10% of *F_max_*.

The stiffness coefficient, *c*, as a deformation characteristic of the corner joint under the compression bending test, is defined as the arithmetic mean of the result of (9) for each test sample when loaded in the section that corresponds to the linear section on the curve of the correlation between the bending moment and the corner deformation of the joint.

The test samples were tested on a Zwick/Roell Z010 universal testing machine (ZwickRoell GmbH & Co. KG, Ulm, Germany) at the University of Chemical Technology and Metallurgy (CCTM) in the Department of Pulp, Paper and Printing. Tests were carried out at a temperature of 20 ± 2 °C and a relative humidity of 55 ± 5%.

### 2.4. Statistical Processing

A descriptive statistical analysis of the results was carried out with XLSTAT statistical and data analysis solution Lumivero (2023) [36]. To detect the presence of extreme outliers in the results obtained in the present study, Grubbs’ test was used to detect outliers in a data set that is assumed to be outside a normally distributed population. In the final statistical processing, all values that were outside the normal distribution were removed. A one-way ANOVA was performed on the results for the bending strength and stiffness coefficient of corner joints to analyse variance at a 95% confidence interval (*p* < 0.05). The statistical differences between mean values were evaluated using Tukey’s honest significant difference (HSD) post hoc test.

## 3. Results

### 3.1. Bending Moments of the Joints

**Figure 7:**
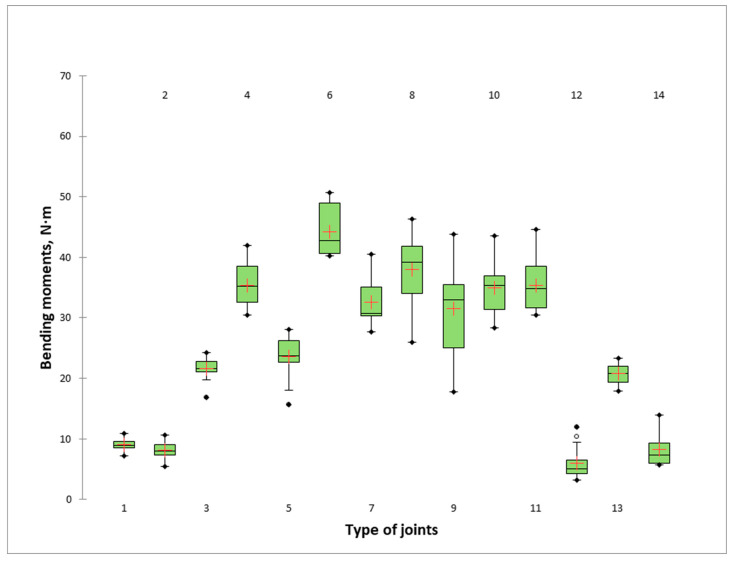
Bending strength under the compression test of L-type mitred corner joints made of thin and ultra-thin structural elements. The number index of the type of joints is based on the data presented in Figure 3.

The results for the bending moment of end-corner joints of thin and ultra-thin materials are given graphically in Figure 7. Table 3 shows the Tukey’s HSD analysis of the differences between the groups with a confidence interval of 95% of bending capacity of mitre joints made of thin and ultra-thin structural elements of all pairwise comparisons. The joints are ranked in descending order of bending moments.

The results obtained for the bending moment of the joints clearly show that it varies over an extensive range. The highest strength (44.23 N·m) is found in the mitre joints made of MDF, laminated with HPL, with a total thickness of 11.6 mm (6_MDF_10_HPL). The lowest bending moment (5.95 N·m) was obtained in the joints made of compact HPL (12_HPL_10_Comp). The difference is seven-and-a-half times, which can be defined as very significant. From the statistical analysis of the one-way ANOVA test and the pairwise comparison performed with the Tukey’s HSD analysis, a significant difference of α = 0.05 at a confidence level of 95% was found in six groups between the obtained bending strength of the L-type end-corner joints constructed from thin and ultra-thin panels. The groups are shown in Table 3. In addition to the joint with the highest strength, six other joints have a bending moment of more than 30 N·m, indicating the extremely high strength of these joints. As expected, the second-best joints in terms of bending strength (37.82 N·m) are the joints made by gluing 12 mm thick plywood panels (8_Ply_12). The next group includes three joints of similar strength: the 10 mm thick (4_MDF_10) joint with a bending moment of 35.37 N·m and the two detachable joints made of MDF (11_MDF_12_M_15) and PB (10_LPB_12_M_15), both 12 mm thick, with bending moments of 35.34 and 34.95 N·m, respectively. The mitre joints made of plywood with a thickness of 9 mm (7_Ply_9) and laminated plywood (9_Ply_9_HPL) also have very good bending strength, with bending moments of 32.61 and 31.51 N·m, respectively. Relatively good bending strengths are achieved in the group of glued mitre joints made of coloured MDF (23.61 N·m), PB (21.59 N·m) and compact HPL joined with Bison Grisly Extreme adhesive (20.84 N·m). The group of joints with the lowest strength include both joints by biscuit in plywood 12 mm thick (9.07 N·m) and in MDF 12 mm thick (8.07 N·m) and both joints by compact HPL with adhesives Bison Montage Kit (8.28 N·m) and with Soudal SoudaBond Easy (5.95 N·m).

**Figure 8:**
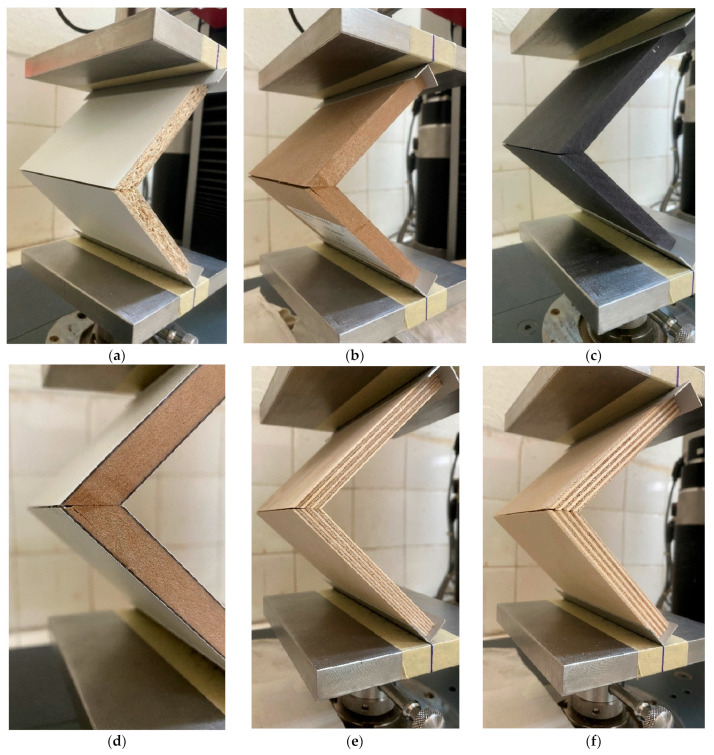
The typical mode of failure of mitre joints under the compression test of L-type mitred corner joints made of thin and ultra-thin structural elements with PVAc adhesive: (**a**)—12 mm LPB (3_LPB_12); (**b**)—10 mm MDF (4_MDF-10); (**c**)—10 mm MDF black (5_MDF_10_Black); (**d**)—laminated 10 mm MDF (6_MDF_10_HPL); (**e**)—9 mm plywood (7_Ply_9); and (**f**)—12 mm plywood (8_Ply_12).

The mode failure of the mitre joints with a biscuit is caused by pulling out the biscuit and partially breaking the panel around the connecting point. The typical mode of failure in all series of mitre joints made of wood-based materials and glued with PVAc adhesive is caused by breaking the adhesive line (Figure 8); in addition to failure at the outer edge, splitting occurs along the thickness of the plate (Figure 8b–d).

**Figure 9:**
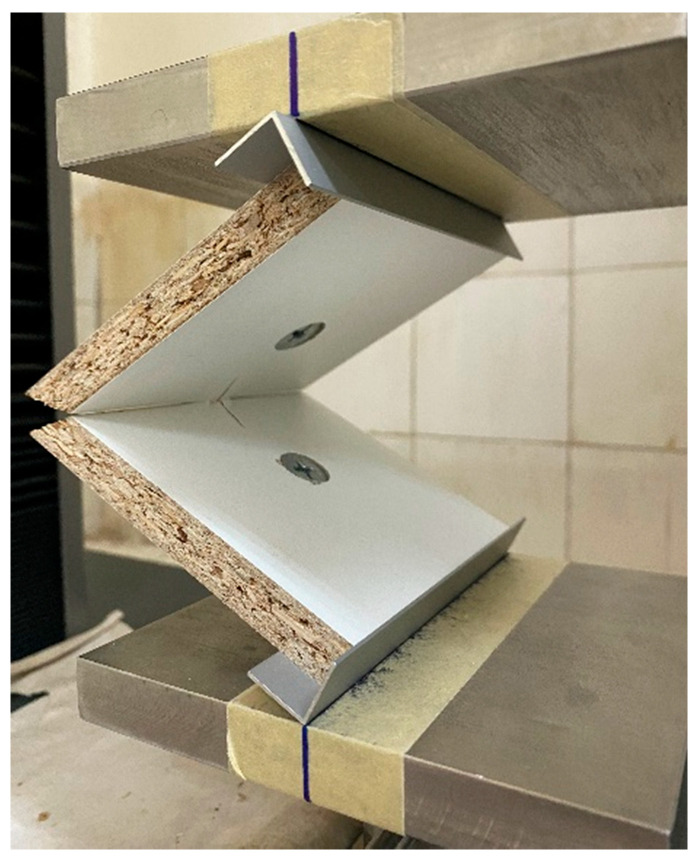
The typical mode of failure under the compression test of L-type mitred corner joints made of LPB and connected with Minifix and a double-ended bolt (10_LPB_12_M_15).

In the case of detachable joints with Minifix and a double-ended bolt (10_LPB_12_M_15 and 11_MDF_12_M15), there is virtually no actual failure of the joint, as it has a resistance moment until the arms finally come together (Figure 9).

The joint made of compact HPL with BISON Grissly Extreme adhesive (13_HPL_10_Comp), which has a very high bending moment (20.84 N·m), does not fail. The adhesive seam is extremely elastic, and even with a significant continuation of the load after the ultimate value is reached, the joint does not fail. It is almost the same situation with mitre joints with the BISON Montage KIT (13_HPL_10_Comp), while in the case of the Soudal SoudaBond Easy adhesive (12_HPL_10_Comp), failure of the joint is complete and occurs suddenly.

### 3.2. Stiffness Coefficients of the Joints

**Figure 10:**
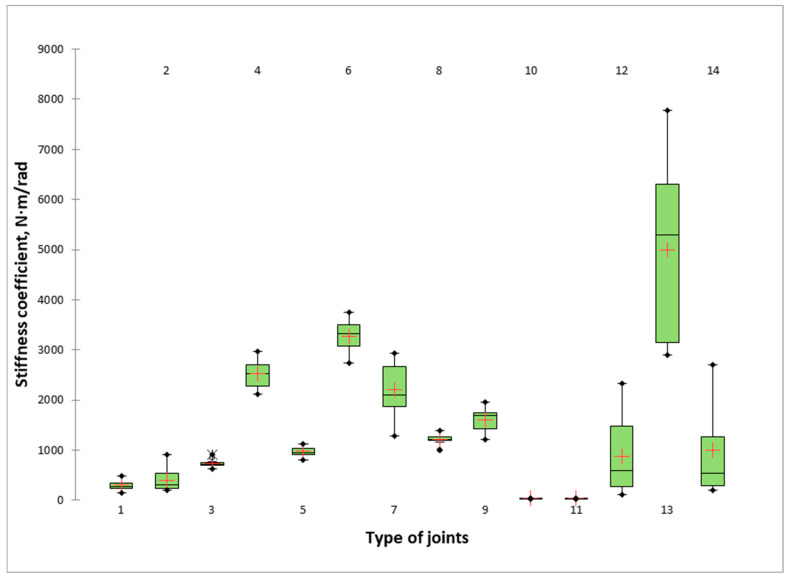
Stiffness coefficients of mitre joints made of thin and ultra-thin structural elements. The number index of the type of joints is based on the data presented in Figure 3.

The results for the stiffness coefficients of L-type mitre joints made of thin and ultra-thin materials are presented graphically in Figure 10. Table 4 shows the results of the Tukey’s HSD analysis of the differences between the groups with a confidence interval of 95% on the bending capacity of mitre joints made of thin and ultra-thin structural elements of all pairwise comparisons. The joints are ranked in descending order of stiffness coefficients.

The situation for stiffness coefficients differs significantly from that for bending moments. The joints with the greatest stiffness (4992.42 N·m/rad) are made of compact HPL (13_HPL_10_Comp) glued with Bison Grisly Extreme. Surprisingly, this joint is the tenth in bending moment and has the highest stiffness, which is higher by just over 50% compared to the joint in second place and the joint with the highest strength (6_MDF_10_HPL). Even more surprising is the result for the stiffness of the two detachable joints by the eccentric connector and double-ended bolt (11_MDF_12_M_15 and 10_LPB_12_M_15), which ranked last in stiffness with coefficients of 35.34 and 34.95 N·m/rad, respectively, and ranked fourth and fifth in bending moments. Joints with excellent stiffness and bending strength are the mitre joints made by gluing MDF with a thickness of 10 mm (4_MDF_10) and the joints of plywood with a thickness of 9 mm (7_Ply_9) and laminated 9 mm thick plywood (9_Ply_9_HPL). Their stiffness ranges from 1607 to 2615 N·m/rad. Mitre joints with Lamello biscuits (2_MDF_12_L0 and 1_Ply_12_L0) have relatively low stiffness, with stiffnesses of 394 and 299 N·m/rad, respectively.

## 4. Discussion

The glued mitre joints and the detachable joints made of thin and ultra-thin wood materials show excellent strength. The compact HPL joint can be added to these. All these joints have bending moments from 44.23 to 20.84 N·m. These values are close to the results obtained in a study of the bending strength under arm compression loading of 15 mm thick plywood joints [19]. 

The results show that lamination of MDF with HPL increases the bending strength and stiffness of the glued mitre joints by 25%. Lamination of 9 mm plywood does not increase the joint’s strength or stiffness. There are contradictory trends in which the characteristics of 9 and 12 mm plywood joints change. As the thickness increases, the bending moment increases by 16%, but the stiffness decreases by 45%. A significant difference was found in the joint properties of the standard MDF and the black-coloured one. The standard one provides the glued mitre joints with a 50% higher bending moment and more than two-and-a-half times higher stiffness. This is probably due to the significantly higher MOE and BS values of standard MDF.

The bending moment results obtained for the glued mitre joints of 12 mm PB can only be compared with those of Norvydas et al. [30], where a very high bending moment of 154 N·m was obtained, which is seven times higher strength than the results in this study. The same authors [30] concluded that the case furniture strength and stiffness are higher by two to four times with mitre joints compared to the other joints.

A significant difference can be seen in comparing the results of Langova and Joscak [15,16] for 12 mm PB joints with different types of screws. In their study, the bending moment is between 3.63 and 11.31 N·m, which is much less than the values obtained for 12 mm PB in this study. The situation is similar to the stiffness of the joints, where the highest stiffness coefficient is 184.92 N·m/rad and the lowest is 73.31 N·m/rad.

We could not find any data in the literature on the strength and deformation characteristics of detachable mitre joints made of thin and ultra-thin materials. Thus, the only possibility of analysing the results obtained is to compare them with the bending moments of joints obtained by Minifix connectors or similar for laminated PB and MDF materials with a standard thickness of 16–18 mm obtained by other authors [37,38,39,40]. They show that the bending moment ranges from 6.02 to 10.62 N·m. The results in the present study are 34.95 and 35.37 N·m, which is an increase of between four and six times in favour of the joint made from the thin material, despite the fact that, in some of the studies, the authors used two [37,38] or four connecting elements [39]. The reason for these high values can be sought in constructing the connecting element itself, namely the double-ended bolt. In practice, it provides a very high bending moment since, to destroy the joint, the entire material must be destroyed, and not just the area of the fastener, as with other joints.

This is not the case with the stiffness of detachable mitre joints. The results obtained are very low and show that, in practice, this type of joint can only be used in furniture structures where stiffness can be ensured by complementary structural elements or connecting elements.

The mitre joints of structural elements of compact HPL show very heterogeneous results regarding bending moment (5.95–20.84 N·m) and stiffness ratio (879–4992 N·m/rad). The serious scatter of results was also observed within a series. This implies that the type of adhesive and its application method play a significant role in the strength and stiffness of the joints. The stiffness of all three types of joints by compact HPL was within a significantly more acceptable range.

## 5. Conclusions

This study proves that thin and ultra-thin wood materials can successfully be used in furniture construction. Compact HPL is also a very suitable material for use, but the choice of adhesive is very important. As a result, the following conclusions were drawn:

1. Materials such as 10 mm thick MDF, laminated MDF, 9 mm plywood, laminated 9 mm plywood and 12 mm plywood are particularly suitable for constructing furniture from thin and ultra-thin materials. Their mitre joints have high strength and deformation characteristics.
2. A laminated PB with a thickness of 12 mm, which is traditionally among the most-used materials for furniture production, also has relatively good strength and deformation characteristics.
3. Glued mitre joints show excellent strength and deformation characteristics and are preferable in furniture made of thin and ultra-thin materials.
4. MDF’s lamination significantly influences mitre joints’ strength and deformation characteristics.
5. Joints with Lamello biscuits must be bonded over the entire edge of the joined elements for higher strength.
6. The stiffness of mitre detachable joints by an eccentric connector with a double-ended bolt is very low, and these joints should be used with caution, even though they have a very high bending strength.
7. The type of adhesive and its application play a significant role in the strength and stiffness of mitre joints of structural elements made of compact HPL. When a very high-stiffness joint is required, joints of compact HPL glued with Bison Grisly Extreme are particularly suitable.

Future research on the possibilities of using thin and ultra-thin wood and non-wood materials can continue to optimise the parameters of the joints and develop a theoretical model to evaluate the properties of these joints.

## Figures and Tables

**Table 1:** Density, thickness, modulus of elasticity and bending strength of thin and ultra-thin structural wood-based and non-wood materials [5].

Material Index *	Density, (D) kg/m^3^	Thickness, (T) mm	Modulus of Elasticity, (MOE) N/mm^2^	Bending Strength, (BS) N/mm^2^
a	703.94	9.13	10,035	103.85
b	821.26	10.73	9039	98.05
c	788.74	10.11	3533	34.97
d	885.77	11.75	5773	51.19
e	756.12	10.25	3105	30.97
f	777.24	12.14	3399	34.97
g	666.71	12.32	9105	86.59
h	708.96	12.14	3815	26.09
i	1490.37	10.45	8886	104.90

* The Material Index is based on the numbering presented in Figure 1.

**Table 2:** Description of end-corner joints made of thin and ultra-thin structural elements.

Number According to Figure 3	Type of the Material	Thickness of the Panels, mm	Face Material	Total Thickness δ, mm	Connecting Element/Adhesive	Index of the Joints
1	Plywood	12	-	12	Lamello L0/PVAc	1_Ply_12 _L0
2	MDF	12	-	12	Lamello L0/PVAc	2_MDF_12 _L0
3	LPB	12	-	12	PVAc	3_LPB_12
4	MDF	10	-	10	PVAc	4_MDF_10
5	MDF black	10	-	10	PVAc	5_MDF_10_Black
6	MDF	10	HPL 0.8	11.6	PVAc	6_MDF_10_HPL
7	Plywood	9	-	9	PVAc	7_Ply_9
8	Plywood	12	-	12	PVAc	8_Ply_12
9	Plywood	9	HPL 0.8	10.6	PVAc	9_Ply_9_HPL
10	LPB	12	-	12	Minifix ø15	10_LPB_12_M_15
11	MDF	12	-	12	Minifix ø15	11_MDF_12_M_15
12	Compact HPL	10	-	10	Soudal SoudaBond Easy	12_HPL_10_Comp
13	Compact HPL	10	-	10	Bison Grisly Extreme	13_HPL_10_Comp
14	Compact HPL	10	-	10	Bison Montage Kit	14_HPL_10_Comp

**Table 3:** Results of the Tukey’s HSD analysis of the differences between the groups with a confidence interval of 95% on the bending capacity of mitre joints made of thin and ultra-thin structural elements of all pairwise comparisons.

Ranking	Index of Joints	LS Means, N·m	Standard Error, N·m	Lower Bound (95%)	Upper Bound (95%)	Homogeneity Groups
1	6_MDF_10_HPL	44.23	1.10	41.87	46.58	A
2	8_Ply_12	37.97	1.51	34.71	41.22	B
3	4_MDF_10	35.37	0.90	33.43	37.30	BC
4	11_MDF_12_M_15	35.34	1.17	32.82	37.87	BC
5	10_LPB_12_M_15	34.95	1.12	32.57	37.33	BC
6	7_Ply_9	32.61	1.08	30.30	34.92	C
7	9_Ply_9_HPL	31.51	1.85	27.54	35.49	C
8	5_MDF_Black	23.61	0.97	21.45	25.72	D
9	3_LPB_12	21.59	0,51	20.48	22.70	D
10	13_HPL_10_Comp	20.84	0.59	19.47	22.20	D
11	1_Ply_12_L0	9.07	0.31	8.39	9.75	E
12	14_HPL_10_Comp	8.28	0.92	6.20	10.36	E
13	2_MDF_12_L0	8.09	0.36	7.31	8.87	E
14	12_HPL_10_Comp	5.95	0.60	4.68	7.22	E

**Table 4:** Results of Tukey’s HSD analysis of the differences between the groups with a confidence interval of 95% on the stiffness coefficients of mitre joints made of thin and ultra-thin structural elements of all pairwise comparisons.

Ranking	Index of the Joints	LS Means, N·m/rad	Standard Error, N·m/rad	Lower Bound (95%), N·m/rad	Upper Bound (95%), N·m/rad	Homogeneity Groups
1	13_HPL_10_Comp	4992.42	581.82	3676.25	6308.60	A
2	6_MDF_10_HPL	3276.87	80.88	3103.40	3450.34	B
3	4_MDF_10	2615.84	85.83	2335.18	2712.98	BC
4	7_Ply_9	2211.96	128.12	1935.17	2488.75	CD
5	9_Ply_9_HPL	1585.94	59.52	1480.09	1735.42	DE
6	8_Ply_12	1219.80	22.77	1170.59	1268.99	EF
7	14_HPL_10_Comp	999.57	373.16	117.18	1881.95	EFG
8	5_MDF_Black	975.46	22.44	927.34	1023.58	EFG
9	12_HPL_10_Comp	879.10	182.68	493.69	1264.51	FG
10	3_LPB_12	726.26	18.89	685.74	766.78	FGH
11	2_MDF_12_L0	393.97	55.67	273.69	514.23	GHI
12	1_Ply_12_L0	299.50	28.13	238.21	360.797	GHI
13	11_MDF_12_M_15	35.34	1.17	32.82	37.87	HI
14	10_LPB_12_M_15	34.95	1.12	32.57	37.33	I

## Data Availability

Not applicable.

## References

[1] Jivkov V., Petrova B. Challenges for Furniture Design with Thin Structural Materials. Proceedings of the VI International Furniture Congress, KTU Trabzon.

[2] Petrova B., Jivkov V. (2022). Thin Panel Furniture—Fashion and Challenges. Part I. Types of Thin Materials, Advantages and Disadvantages of their Use. DMT—Design, Materials, Technologies, (XXIV).

[3] Jivkov V., Elenska-Valchanova D. Mechanical Properties of Some Thin Furniture Structural Composite Materials. Proceedings of the 30th International Conference on Wood Science and Technology (ICWST), 70th Anniversary of Drvna industrija Journal, Implementation of Wood Science in Woodworking Sector.

[4] Barboutis I., Kamperidou V. (2011). Properties of Two Different Thickness 3-Ply Plywood of Tree-of-Heaven Veneers. Proceedings of the 22nd International Scientific Conference Wood Is Good—EU Preaccession Challenges of the Sector.

[5] Jivkov V., Petrova B., Yavorov N. Comparative Analysis of Physical and Mechanical Properties of Some Thin and Ultra-Thin Wood-Based and Non-Wood-Based Furniture Panels. Innovation in Woodworking Industry and Engineering Design. Proceedings of the Eleventh International Scientific and Technical Conference “Innovations In Forest Industry and Engineering Design” Inno 2022.

[6] Fekiač J., Svoreň J., Gáborík J., Němec M. (2022). Reducing the energy consumption of circular saws in the cutting process of plywood. Coatings.

[7] Vasileiou V., Kamperidou V., Barboutis I. (2011). Properties of thin 3-ply plywood constructed with tree-of-heaven and poplar wood. Proceedings of the International Conference ICWSE 2011 “Wood Science and Engineering in the Third Millennium”, Brasov, Romania, 3–5 November 2011.

[8] Zhou J., Hu C., Hu S., Yun H., Jiang G., Zhang S. (2012). Effects of temperature on the bending performance of wood-based panels. BioResources.

[9] Sala C.M., Robles E., Gumowska A., Wronka A., Kowaluk G. (2020). Influence of Moisture Content on the Mechanical Properties of Selected Wood-based Composites. BioResources.

[10] Antov P., Valchev I., Savov V. Experimental and statistical modeling of the exploitation properties of eco-friendly MDF through variation of lignosulfonate concentration and hot pressing temperature. Proceedings of the 2nd International Congress of Biorefinery of Lignocellulosic Materials (IWBLCM2019).

[11] Pinchevska O., Zavorotnuk O., Spirochkin A., Sedliačik J., Hrabar I., Oliynyk R. (2021). Durability of kitchen furniture made from medium-density fibreboard (MDF). Acta Fac. Xylologiae Zvolen.

[12] Savov V., Valchev I., Antov P. Processing Factors Affecting the Exploitation Properties of Environmentally Friendly Medium Density Fibreboards Based on Lignosulfonate Adhesives. Proceedings of the 2nd International Congress of Biorefinery of Lignocellulosic Materials (IWBLCM2019).

[13] Savov V., Antov P. (2020). Engineering the Properties of Eco-Friendly Medium Density Fibreboards Bonded with Lignosulfonate Adhesive. Drv. Ind. Znan. Časopis Za Pitanja Drv. Tehnol..

[14] Segovia F., Blanchet P., Barbuta C., Beauregard R. (2015). Aluminum-laminated panels: Physical and Mechanical Properties. BioResources.

[15] Langova N., Joscak P. (2018). Mechanické Vlastnosti Skrutkových Spojov Nábytkových Konštrukcií.

[16] Langova N., Joščák P. (2019). Mechanical properties of Confirmat screws corner joints made of native wood and wood-based composites. Ann. Wars. Univ. Life Sci. SGGW For. Wood Technol..

[17] Máchová E., Langová N., Réh R., Joščák P., Krišťák Ľ., Holouš Z., Igaz R., Hitka M. (2019). Effect of moisture content on the load carrying capacity and stiffness of corner wood-based and plastic joints. BioResources.

[18] Yuksel M., Yildirim N., Kasal A., Erdil Y.Z., Demirci S. (2014). Effect of the panel type and panel thickness on moment resistance of screw-jointed corner joints and stiffness of four-member cabinets. BioResources.

[19] Yuksel M., Kasal A., Erdil Y.Z., Acar M., Kuşkun T. (2015). Effects of the panel and fastener type on bending moment capacity of L-type joints for furniture cases. Pro Ligno.

[20] Jivkov V. (2002). Strength Characteristics of Detachable End Corner Joints Made of 12 and 16 mm Thick Panels by “Minifix” under Bending Loading, no. 1.

[21] Atar M., Ozcifci A., Altinok M., Celikel U. (2009). Determination of diagonal compression and tension performances for case furniture corner joints constructed with wood biscuits. Mater. Des..

[22] Eckelman C.A., Erdil Z. Joints design manual for furniture frames constructed of plywood and oriented strand board. Proceedings of the 1st International Furniture Congress and Exhibition.

[23] Eckelman C.A., Erdil Z. (2000). Joint Design Manual for Furniture Frames Constructed of Plywood and Oriented Strand Board.

[24] Kociszewski M. (2005). Stiffness and load capacity of biscuit corner joints. Folia For. Pol..

[25] Tankut A.N., Tankut N. (2004). Effect of some factors on the strength of furniture corner joints constructed with wood biscuits. Turk. J. Agric. For..

[26] Tankut A.N., Tankut N. (2009). Investigations the effects of fastener, glue, and composite material types on the strength of corner joints in case-type furniture construction. Mater. Des..

[27] Vassiliou V., Barboutis I. Tension strength of furniture middle joints constructed with biscuits. Proceedings of the 10th Anniversary Conference of Engineering Design, Interior and Furniture Design.

[28] Kyuchukov G., Jivkov V. (2016). Furniture Construction. Structural Elements and Furniture Joints.

[29] Maleki S., Haftkhani A.R., Dalvand M., Faezipour M., Tajvidi M. (2012). Bending moment resistance of corner joints constructed with spline under diagonal tension and compression. J. For. Res..

[30] Norvydas V., Baltrušaitis A., Juodeikienė I. (2012). Investigation of miter corner joint strength of case furniture from particleboard. Mater. Sci..

[31] Petrova B., Jivkov V. (2022). Furniture from Thin Structural Elements. Part II. Furniture from Non-Wood Materials. DMT—Design, Materials, Technologies, (XXIV).

[32] Šipailaitė-Ramoškienė V., Fataraitė E., Mickus K.V., Mažeika R. (2003). The Adhesion, Mechanical Properties and Water Resistance of Vinyl Acetate Copolymer Based Blends. Mater. Sci. (Medžiagotyra).

[33] (2013). Thermal Insulating Products for Building Applications—Determination of Tensile Strength Perpendicular to Faces.

[34] (2013). Thermal Insulating Products for Building Applications—Determination of Shear Behaviour.

[35] Jivkov V., Marinova A. Investigation on ultimate bending strength and stiffness under compression of corner joints from particleboard with connectors for DIY furniture. Proceedings of the International Scientific Conference Interior and Furniture Design.

[36] https://www.xlstat.com/en.

[37] Krzyżaniak Ł., Smardzewski J. (2019). Strength and stiffness of new designed externally invisible and demountable joints for furniture cases. Eng. Struct..

[38] Jivkov V., Grbac I. Influence of the cyclic loading on bending strength of different end corner joints made of MDF. Proceedings of the 22nd International Scientific Conference “Wood Is Good—EU Preaccession Challenges of the Sector”.

[39] Simek M., Haviarova E., Slaven I. (2008). Cam Connectors Stiffness and Load Capacity of Furniture Corner Joints. Symposium Furniture 2008.

[40] Joscak P., Cernok A. (2002). Load-Carrying Capacity of Demountable Furniture Joints of Chipboards.

